# An efficient and broadly applicable method for transient transformation of plants using vertically aligned carbon nanofiber arrays

**DOI:** 10.3389/fpls.2022.1051340

**Published:** 2022-11-23

**Authors:** Jessica M. Morgan, Joanna Jelenska, Dale Hensley, Scott T. Retterer, Jennifer L. Morrell-Falvey, Robert F. Standaert, Jean T. Greenberg

**Affiliations:** ^1^ Biophysical Sciences, The University of Chicago, Chicago, IL, United States; ^2^ Department of Molecular Genetics and Cell Biology, The University of Chicago, Chicago, IL, United States; ^3^ Center for Nanophase Materials Science, Oak Ridge National Laboratory, Oak Ridge, TN, United States; ^4^ Biosciences Division, Oak Ridge National Laboratory, Oak Ridge, TN, United States; ^5^ Department of Chemistry, East Tennessee State University, Johnson City, TN, United States

**Keywords:** transient plant transformation, nanomaterials, vertically aligned carbon nanofibers (VACNF), impalefection, fluorescence imaging

## Abstract

Transient transformation in plants is a useful process for evaluating gene function. However, there is a scarcity of minimally perturbing methods for gene delivery that can be used on multiple organs, plant species, and non-excised tissues. We pioneered and demonstrated the use of vertically aligned carbon nanofiber (VACNF) arrays to efficiently perform transient transformation of different tissues with DNA constructs in multiple plant species. The VACNFs permeabilize plant tissue transiently to allow molecules into cells without causing a detectable stress response. We successfully delivered DNA into leaves, roots and fruit of five plant species (Arabidopsis, poplar, lettuce, *Nicotiana benthamiana*, and tomato) and confirmed accumulation of the encoded fluorescent proteins by confocal microscopy. Using this system, it is possible to transiently transform plant cells with both small and large plasmids. The method is successful for species recalcitrant to *Agrobacterium*-mediated transformation. VACNFs provide simple, reliable means of DNA delivery into a variety of plant organs and species.

## 1 Introduction

The ability to introduce DNA into plants and achieve plant transformation has radically changed agriculture and provided a means to study fundamental processes in plants. Transformations can involve the integration of DNA into a plant genome (stable transformation) or the introduction of DNA into plant cells without the incorporation of transgenes into the genome (transient transformation) (Altpeter et al., 2016). Additionally, plastids, cellular organelles in plants with their own genomes, can be stably and transiently transformed (Maliga, 2004; Yu et al., 2020). Transient transformation is faster than stable transformation. It provides the opportunity to study the functions and regulation of genes either in single cells or within the context of plant organs (Birch, 1997; Sheen, 2001; Krenek et al., 2015). For example, reporters can be introduced to monitor transcriptional activation, the subcellular localization of proteins, and subcellular processes through the delivery of different molecular probes (Levy et al., 2018).

Three of the main methods employed for transient transformation in plants involve *Agrobacteria*, protoplast transfection, and particle bombardment. *Agrobacterium*-mediated transformation is limited to plant species, cultivars, and tissue types (roots, leaves, and seedlings) that are susceptible to this bacterium (Hwang et al., 2017). High cell densities of *Agrobacteria* are required to perform transient transformation (agroinfiltration), which can be harmful to plants (Karami, 2008; Li et al., 2009). For protoplast transfection, protoplasts are made *via* enzymatic breakdown of the cell wall after grinding up tissue. DNA is then delivered to the protoplasts *via* electroporation or polyethylene glycol using small vectors that are easy to manipulate. This process is advantageous because transient transformation can be achieved with a high frequency. Like *Agrobacterium*-mediated transformation, however, protoplast transfection is labor intensive, it requires optimization for each species (Baltes et al., 2017; Gou et al., 2020; Ren et al., 2020), and it does not allow studies of intact tissues. Finally, particle bombardment can be used to deliver DNA into a broader range of plants and tissues than agroinfiltration and protoplast transformation. The tissues to which particle bombardment can be applied include meristems, embryos, leaves, callus, stems, roots, pollen, styles, and petals (Lacroix and Citovsky, 2020). Particle bombardment, however, causes plant tissue damage when high bombardment pressures are used. Other limitations arise from the need to position plant material in the biolistic chamber or to align it with a gene gun, which limit the size of the specimen and the accessible sites (Haque et al., 2018; Mohan et al., 2019; Ozyigit and Yucebilgili Kurtoglu, 2020). Notably, all these methods affect plant physiology by inducing stress responses that may interfere with subsequent experiments using transiently transformed cells.

Nanomaterials are being developed for the delivery of biomolecules into plants because of their applicability to several different plant species and for their ability to increase transformation efficiency *in planta* (Kumar et al., 2020). The use of nanomaterials as vehicles for biomolecules has been widely studied in animal cells and tissues for drug delivery, but not in plants (McKnight et al., 2004; McKnight et al., 2005; Cunningham et al., 2018; Sanzari et al., 2019; Jain and Thareja, 2019; Jat et al., 2020). This may be due in part to the barriers presented by the cell wall and the fact that different plant species have varying physiologies, which need to be considered when addressing the interactions between the host environment and the nanomaterial (Pérez-de-Luque, 2017). To overcome the hurdles presented by plants, the size of the nanoparticles has been tuned, and surface modifications have been made to enable attachment of cargo to nanovehicles (Kumar et al., 2020). Some examples of these nanovehicles include: surface functionalized and pristine single walled carbon nanotubes (Liu et al., 2009; Demirer et al., 2019), mesoporous silica nanoparticles (MSNs) (Torney et al., 2007; Chang et al., 2013), gold nanoparticles and nanoparticles made of other materials coated with gold, polymeric nanoparticles (Zhang et al., 2019b), DNA nanostructures (Zhang et al., 2019), layered double hydroxide clay nanosheets (Mitter et al., 2017), magnetic nanoparticles (used for pollen magnetofection (Zhao et al., 2017)), silicon carbide whiskers (Arshad et al., 2013), and peptides as carriers for nucleic acids (Zheng et al., 2017; Jat et al., 2020). Of the nanovehicles used to deliver genes/DNA into plants, acicular shaped materials (carbon nanotubes) account for a large portion of the studies conducted. These materials have been delivered into plants through passive delivery, a needleless syringe, or with the aid of ultrasound, a magnetic field, or a gene gun (Jat et al., 2020).

A promising approach used for delivering biomolecules into cultured animal cells was the application of vertically aligned carbon nanofibers (VACNFs; McKnight et al., 2004; McKnight et al., 2005). Since these fibers are similar in scale to aphid stylets but with closed tips, they were assessed by Davern et al. (2016) for their potential to permit delivery of different sized molecules such as dyes, proteins, and dextrans to plant leaves. For this method, molecules dissolved in water were pipetted onto a leaf surface, and overlayed with the fiber array that was then gently tapped to promote penetration of the nanofibers and molecules into the leaf tissue. Importantly, the VACNFs enabled delivery of a variety of molecules into leaf tissue without causing a detectable stress response (Davern et al., 2016). Furthermore, fibers broke away from the backing and remained embedded in cells without causing loss of cell viability, providing markers for which cells potentially received molecules of interest.

In this work, we have pioneered the use of VACNF arrays for the purpose of delivering DNA-encoded probes to plants to enable transient transformation (impalefection). We show the flexibility of the method by using it to transiently transform plant organs in several species with DNA encoding fluorescent proteins that can be detected by confocal microscopy. By examining the plant with a minimally invasive method, this approach will provide a more physiologically accurate picture of signaling pathways than most other studies that rely on detached tissues.

## 2 Materials and methods

### 2.1 Plants

*Arabidopsis thaliana* (Columbia-0 accession) plants 3-4 weeks post vernalization were used for leaf transformations and grown in long day conditions (16 h of light, 8 h of dark) in soil at 19-20°C, 35-75% relative humidity (RH), and 150-200 μmol m^−2^ s^−1^ light in a growth room. Seeds were vernalized in the dark at 4°C for 4 d prior to planting. *Populus trichocarpa* (Nisq-1 genotype) (poplar) plants were grown in soil in long day conditions at 30-60% RH, 150-200 μmol m^−2^ s^−1^ light intensity, 22°C in a greenhouse. Lettuce (*Lactuca sativa* cultivar Salinas) was grown in long day conditions at 19°C in a greenhouse. Second to fourth true leaves of young (20-30 day old) lettuce plants were used for transformation. For experiments with roots, lettuce seeds were germinated at 22°C (long day conditions) for 7-14 days in petri dishes with filter paper and 0.5 g/L MaxiGro (General Hydroponics). *Nicotiana benthamiana* was grown for 4-5 weeks in long day conditions at 24°C in a growth chamber. Tomato fruits, strawberries, apples, and peaches were purchased from a grocery store and were stored at room temperature prior to use. Roots used for VACNF delivery experiments came from Arabidopsis seedlings 5-6 d post planting grown on ½ MS plates supplemented with vitamins, grown under long day light conditions. Seeds for Arabidopsis seedlings were sterilized (70% ethanol for 5 min, 50% chlorine laundry bleach supplemented with 0.5% Tween-20 for 10 min, and washed 4x with dH_2_O) and vernalized in the dark at 4°C for 4 d prior to planting.

### 2.2 Production of the vertically aligned carbon nanofibers

**Figure 1:**
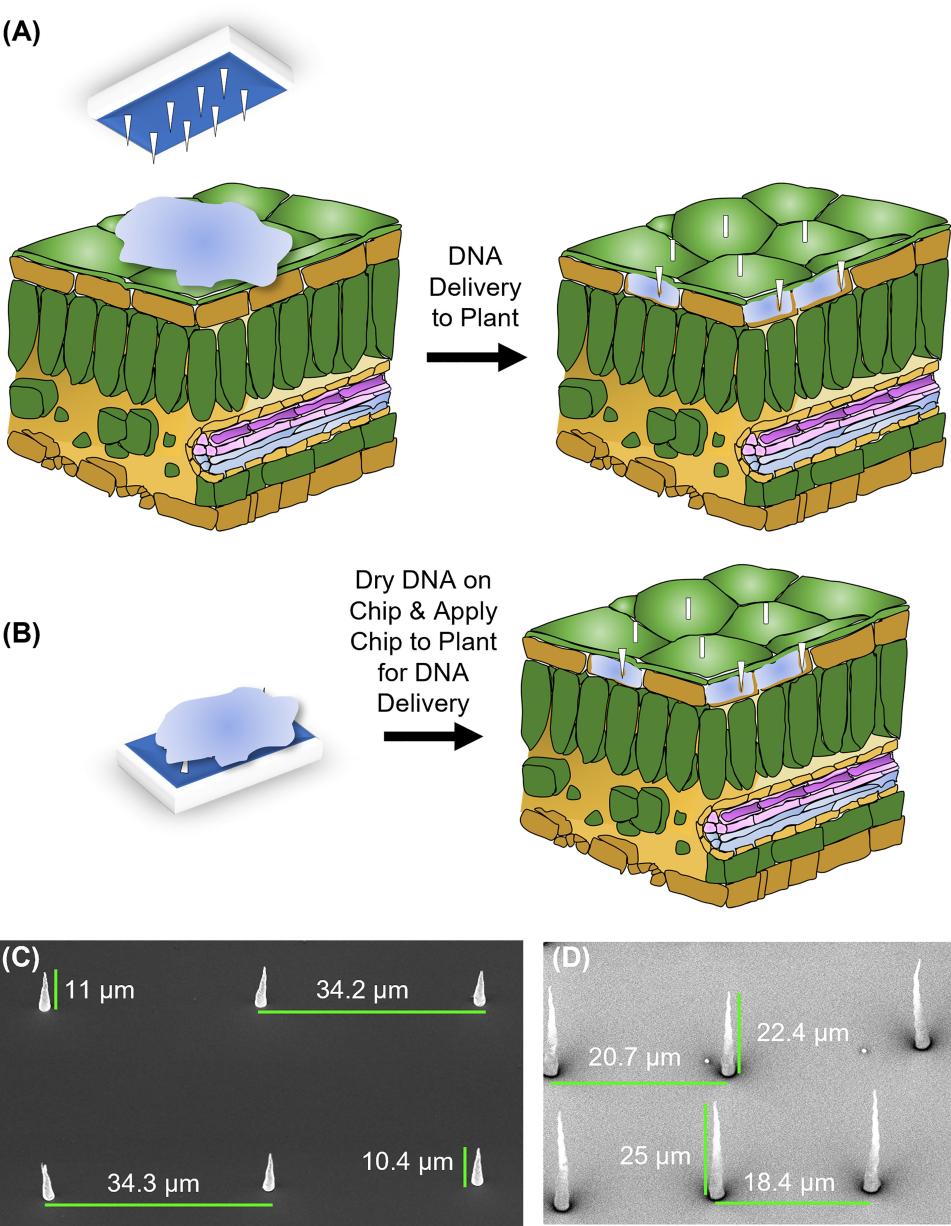
Design and application of carbon nanofibers. **(A)** On-plant of fiber delivery. A chip, placed on top of a leaf on which a 1 μL droplet of DNA solution is placed, is gently tapped to push the fibers into the leaf tissue. The chip is then removed, and nanofibers typically remain embedded in the tissue. **(B)** On-chip DNA method of fiber delivery. Fifteen minutes after a 1 μL drop of DNA is placed on a chip, it is placed on a plant organ surface and tapped with tweezers as in **(A)**. After tapping, the chip was removed, and nanofibers were left embedded in the tissue. For both methods, −fiber controls utilized the flat (fiberless) side of the chip. **(C)** Electron micrograph of VANCFs with ~35 µm pitch, 10-15 µm height, and imaged at a 30° angle. **(D)** Electron micrograph of VANCFs with ~20µm pitch, 20-30 µm height, and imaged at a 30° angle. Due to the fact that the fibers were imaged at 30° angle, the apparent heights appear to be smaller than the actual height by a factor of sin(30°)=1/2. Fibers were grown using 200-500 nm e-beam dot sizes and taper to a diameter less than 200 nm. Growth parameters: plasma current 1 amp, C2H2: NH3 = 0.55, 625°C, 10 torr.

Carbon-based nanofibers with a lateral spacing (pitch) of 10, 20, or 35 μm and height ranging from 15-25 μm were made in the Center for Nanophase Material Science (CNMS) at Oak Ridge National Laboratory using nickel catalyst dots patterned across the top of a silicon wafer *via* electron beam lithography, metal evaporation, and conventional lift-off using the method described by (Melechko et al., 2005; Melechko et al., 2009). For the lithography step, the silicon wafers were coated with polymethyl methacrylate resist and exposed using a JEOL 9300FS electron beam lithography system and developed in a solution of 1:3 methyl-isobutyl ketone: isopropyl alcohol (Nano MIBK/IPA 1:3 Developer, MicroChem, Newton, MA). Electron beam lithography was necessary to define the catalyst dots with diameters between 200 nm and 500 nm. After developing the resist and cleaning with a 6-second exposure to oxygen plasma, electron beam evaporation was used to deposit a thin nickel film (100-125 nm) on the patterned resist. Sequential sonication in acetone and isopropyl alcohol was used to remove the underlying resist layer, leaving catalyst dots in the desired pattern behind. Nanofiber growth was then conducted using a direct current plasma-enhanced chemical vapor deposition chamber using an acetylene/ammonia mixture. Growth parameters were optimized to control the length and taper of the nanofibers (tapering less than 200 nm). After fabricating the VACNFs, their geometry was assessed using scanning electron microscopy (Zeiss Merlin FE-SEM) at a 30° tilt with an acceleration potential of 1 kV (**Figure 1**). To protect VACNF chips during transport and storage, the fibers were coated with a layer of photoresist (SPR955, spun at 1K for 45 s) (Davern et al., 2016). Photoresist was removed *via* increment washes of acetone (100%, 5 min), isopropyl alcohol (100%, 5 min), and water (5 min) prior to use.

### 2.3 DNA and dye delivery *via* VACNFs

#### 2.3.1 Application to leaves, roots, and fruit “on-plant method”

Fiber delivery of DNA or dye to plant cells was conducted using a method modified from Davern et al. (2016). In brief, a 1 μL droplet containing fluorescein dye (1 mM), TAMRA dye (5(6)-Carboxytetramethylrhodamine, 10 μM), or of 200 ng/µl of plasmid DNA purified from liquid bacterial cultures was applied to the surface of a leaf or fruit. For Arabidopsis and poplar leaves, abaxial surfaces of detached leaves were impaled. For lettuce and *N. benthamiana*, adaxial surfaces of leaves attached to plants were impaled. A VACNF chip (3x3 mm) was placed on top of the droplet (with the fibers oriented such that they came into contact with the droplet), and the fibers were tapped into the plant tissue with a pair of forceps, using a hard surface to support the other side of the plant organ. For control experiments, the VACNF chip was applied with its smooth back to the leaf or fruit. Fibers applied without DNA or dye were another control.

For dye delivery experiments, chips were left on the plants for 5 minutes and removed just before imaging. Chips were applied to roots of intact lettuce seedlings and roots were imaged 5 min – 1 h after dye delivery. After VACNF-mediated DNA delivery, Arabidopsis or Populus leaves were stored in a humid chamber for 48 h in long-day conditions (16 h of light, 8 h of dark) prior to imaging. Tomato fruits were kept in long day conditions as well for 40 h after DNA delivery. Arabidopsis leaves transformed with AALP:GFP were stored in the dark for 48 h prior to imaging (Tamura et al., 2003) unless otherwise specified. Lettuce plants were maintained in long day condition for 4 d after impalefection (the period effective for transient transformation *via Agrobacteria* (Wroblewski et al., 2005) and *N. benthamiana* plants for 3 d. Just before imaging, the plant tissues that were in contact with the chips were excised and placed on glass slides. In the case of the tomato fruit, the epidermal layer was removed to expose the top layer of the pericarp. Both the epidermal layer and the pericarp were examined for fibers and fluorescent protein accumulation.

#### 2.3.2 Application to Arabidopsis leaves and roots “on-chip method”

When applying the chips to the Arabidopsis leaves using the “on-chip” method, 1 μL of 200 ng/μL DNA solution was drop-cast on to the fiber side of the chip and allowed to dry for 15 min prior to impaling tissue. When placing the droplet on the fibers, great effort was taken to ensure that the droplet was placed in the center of the chip and covering several fibers. Leaves were placed on a hard substrate and the fibers were applied on the abaxial side of the leaves using a pair of tweezers for the tapping. After VACNF-mediated delivery, plant leaves were stored in a humid chamber for 48 h in long-day conditions. For roots, the same method of drying DNA on VACNFs was applied. Roots were placed on agar plates and chips were applied to the organs while they were on top of the agar. Seedlings with roots transformed with biosensor FlincG (fluorescence indicator of cGMP) *via* VACNFs were kept on agar plates in long day light (16 h light, 8 h dark) conditions for 36 h prior to imaging.

### 2.4 *Agrobacterium*-mediated transformation

Lettuce (cultivar Salinas) and *N. benthamiana* leaves were infiltrated with *Agrobacterium tumefaciens* GV3101 carrying p35S-mCherry/pCambia (Kang et al., 2014) at OD_600_ = 0.6. Plants were grown in the same way as for VACNFs experiments and kept in their growth conditions for 4 d post infection for lettuce and 3 d post infection for *N. benthamiana* before imaging.

### 2.5 Plasmids

The binary ~11,000 bp plasmid pUbiquitin_10_:YFP-Gateway from Michniewicz et al. (2015), the ~3000 bp protoplast vector pUC with p35S:Arabidopsis Aleurain Like Protein (AALP):GFP from Kang et al. (2014), and the ~3000 bp vector pUC with p35S:mCherry, were used for VACNF-mediated transformation. pCambia with identical p35S:mCherry cassette (~10,000 bp) was used for *Agrobacterium*-mediated transformation (Kang et al., 2014). The binary vector pART: delta-FlincG (N2105633 Nottingham Arabidopsis Stock Centre), 6857 bp, with the cGMP biosensor FlincG (Isner and Maathuis, 2011) was also used.

### 2.6 Treatment of seedlings with Pep1

40 μL of Pep1 peptide (20 nM, Biomatik) was added to a glass slide on top of the root prior to imaging to trigger cGMP production in seedlings transiently transformed with the FlincG sensor.

### 2.7 Microscopy

Images were captured with Zeiss LSM 800 or LSM 710 confocal microscope. GFP and fluorescein signals were collected with an emission range of 510 and 545 nm, excitation was 488 nm. YFP signal was collected with an emission range of 491-573 nm, excitation was 488 nm. mCherry was imaged with 561 nm excitation and 570-628 emission.

### 2.8 Integrated density measures for fluorescent proteins

Fluorescence values for AALP:GFP, mCherry, and YFP were measured as total fluorescence (integrated density) in 20 μm × 20 μm confocal image areas (Jelenska et al., 2017) and quantified using ImageJ/FIJI (Schindelin et al., 2012).

### 2.9 Statistical analysis

Statistical analysis (ANOVA and Tukey’s test) was performed using PRISM 9 (GraphPad). Statistically different categories (P<0.05) are marked by different letters.

## 3 Results

### 3.1 Fiber design and application

Two approaches for introducing plasmid DNA into plant cells using VACNF chips were implemented: “on-plant” and “on-chip” methods (**Figures 1A, B**). For the on-plant method, a 1 µl droplet containing DNA was placed on the surface of a leaf. A VACNF chip was orientated such that the fibers came into contact with the droplet. Tweezers were then used to apply a force on the back side of the chip, driving the fibers into the tissue and delivering materials into the plant (**Figure 1A**). With the on-chip method, DNA was first applied to a chip, which was then used to impale the tissue (**Figure 1B**). In this work, we used 200 ng of plasmid DNA in 1 µL of water, after preliminary experiments using different concentrations and the on-plant method suggested this amount was optimal. Our conditions were similar to those used for successfully introducing DNA into mammalian cells using VACNF chips (McKnight et al., 2004; McKnight et al., 2005).

**Figure 2:**
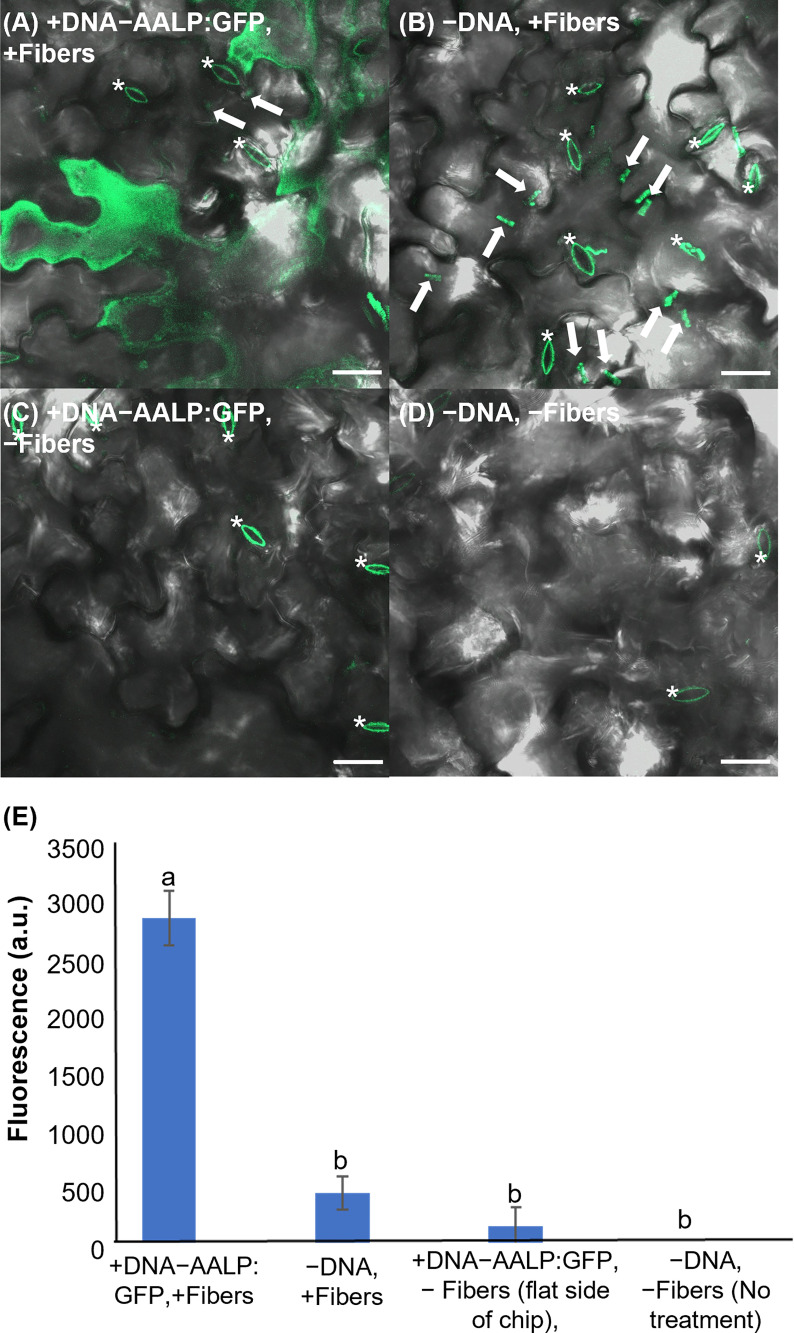
Fluorescent image analysis of Arabidopsis leaves transiently transformed with AALP:GFP 48 h after delivery *via* VACNF with a 35 µm pitch. **(A)** p35S:AALP:GFP/pUC plasmid delivery *via* VACNF using the on-plant method. GFP fluorescence is shown in green (+DNA-AALP:GFP, +fibers). **(B)** Fibers only (−DNA, +fibers). **(C)** AALP:GFP plasmid delivery without fibers (backside of chip with no fibers; +DNA-AALP:GFP, −fibers). **(D)** No treatment (−DNA, −fibers). **(E)** Graph of average integrated densities of GFP fluorescence (green channel) of defined 20×20 μm areas (n≥40 per condition, combined results from two experiments). Regions containing stomata (*) were excluded due to autofluorescence, and the average fluorescence signal intensity from the no-treatment condition was subtracted from each average. 2-way ANOVA (and Tukey test) was used for significance testing, and error bars represent the standard error of the mean. Different letters show significant differences between treatments (*P* < 0.0001). All images shown are maximum projections of 40 μm z-stacks. Scale bars are 20 μm and white arrows indicate fibers in the images.

**Figure 3:**
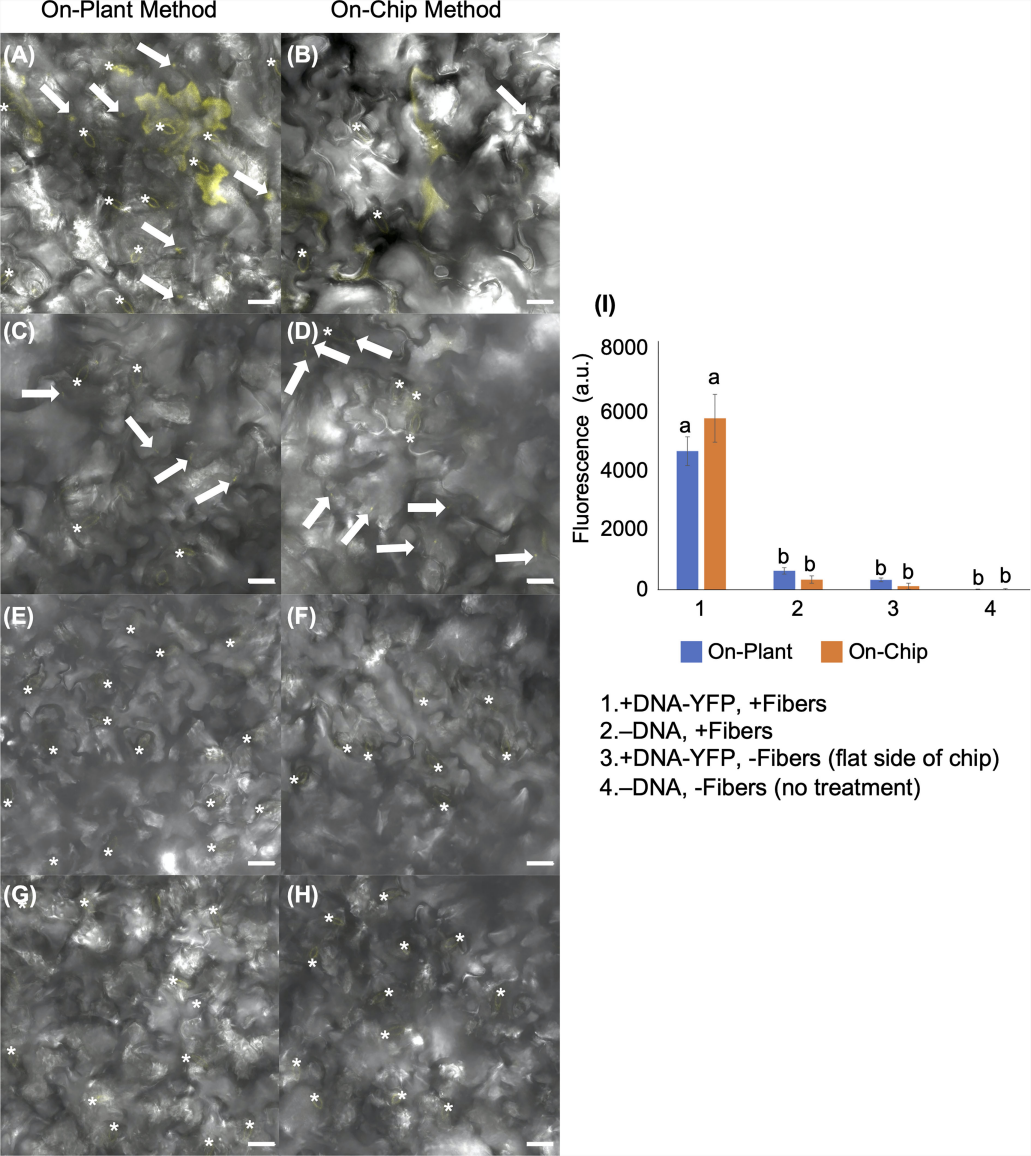
Fluorescent image analysis of Arabidopsis leaves transiently transformed with pUBQ_10_:YFP 48 h after delivery *via* VACNF with a 35 µm pitch using the on-plant or on-chip methods. **(A)** and **(B)** DNA-YFP delivery *via* VACNF using the on-plant and on-chip methods, respectively (+DNA-YFP, +fibers). **(C)** and **(D)** Fibers only (–DNA, + fibers). **(E)** and **(F)** DNA-YFP delivery without fibers (flat chip: +DNA-YFP, −fibers). **(G)** and **(H)** No treatment (−DNA, −fibers). **(I)** Graph representing relative average fluorescence signal intensity of 25 20×20 μm areas from images of 5 biological replicates combined from 2–3 experiments using fluorescence from the YFP channel. Regions containing stomata (*) were excluded due to autofluorescence, and the average fluorescence intensity from the no-treatment condition was subtracted from each average. 2-way ANOVA (and Tukey test) was used for significance testing, and error bars represent the standard error of the mean. Different letters show significant differences between treatments (*P* < 0.0001). All images shown are maximum projections of 40 μm z-stacks. Scale bars are 20 μm, and white arrows indicate fibers in the images.

**Figure 4:**
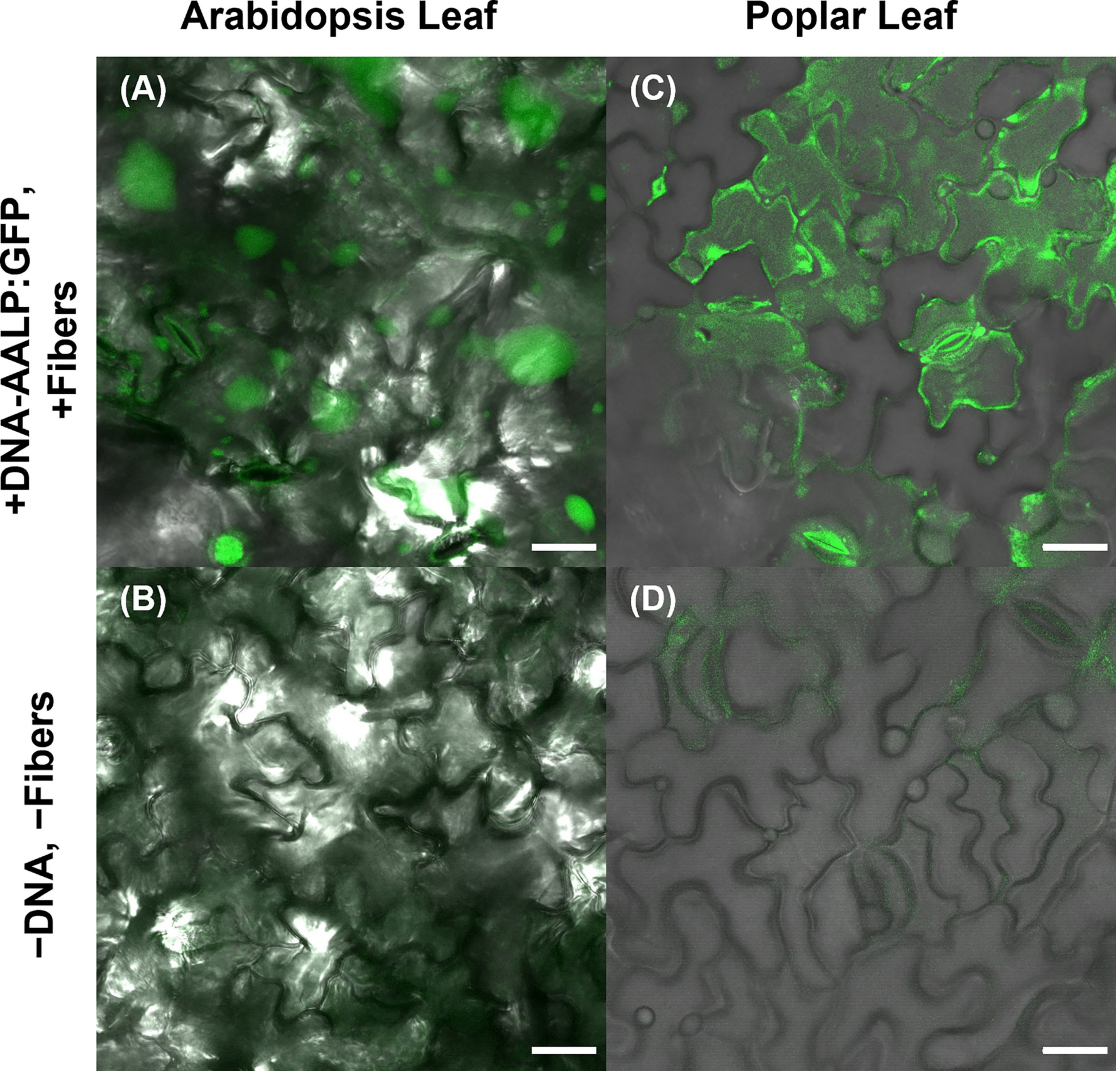
Use of VACNF to introduce DNA expression constructs into poplar leaves. Images were generated using confocal fluorescence microscopy. VACNFs delivered a small plasmid resulting in expression of a vacuolar marker, AALP:GFP (shown in green). **(A)** Arabidopsis leaf 48 h after delivery of p35S:AALP:GFP/pUC *via* fibers with 20 µm pitch (+DNA-AALP:GFP, + fibers), **(C)** Poplar leaf 48 h after delivery *via* fibers with 10-µm pitch (+DNA-AALP:GFP, +fibers). **(B, D)** Show untreated controls (−DNA, −fibers). All images are maximum projections of 40 μm z-stacks. Scale bar = 20 µm.

**Figure 5:**
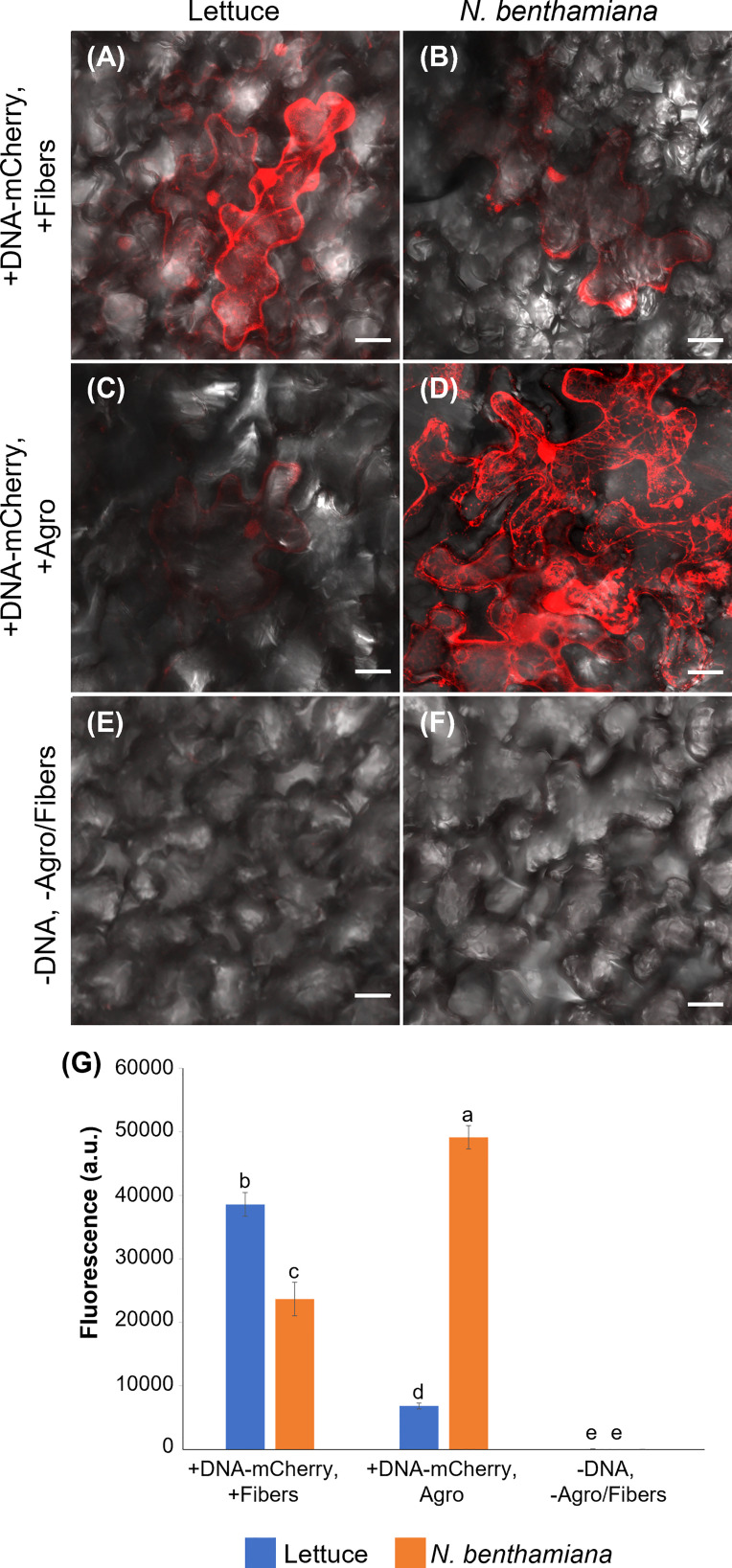
Comparison of VACNF- and *Agrobacterium*-mediated transient transformation. **(A)** p35S:mCherry/pUC expression 4 d after delivery *via* VACNFs with 10 µm pitch using the on-plant method in lettuce and **(B)** 3 d after delivery in *N. benthamiana*. **(C)** *Agrobacterium*-mediated delivery of p35S:mCherry/pCambia in lettuce, 4 d after agroinfiltration. **(D)** p35S:mCherry/pCambia 3 d after agroinfiltration in *N. benthamiana*. **(E)** and **(F)** show background signal for no treatment samples in lettuce and *N. benthamiana*, respectively. **(G)** Graph representing average fluorescence signal intensity of the mCherry fluorescence of 20×20 μm areas (n≥40, combined results from 2–3 experiments). Background signal (no treatment) was subtracted, 2-way ANOVA (and Tukey test) was used for significance testing, and error bars represent the standard error of the mean. Different letters show significant differences between treatments (*P* < 0.0002). All images are maximum projections of 20 μm z-stacks. Scale bars are 20 μm.

**Figure 6:**
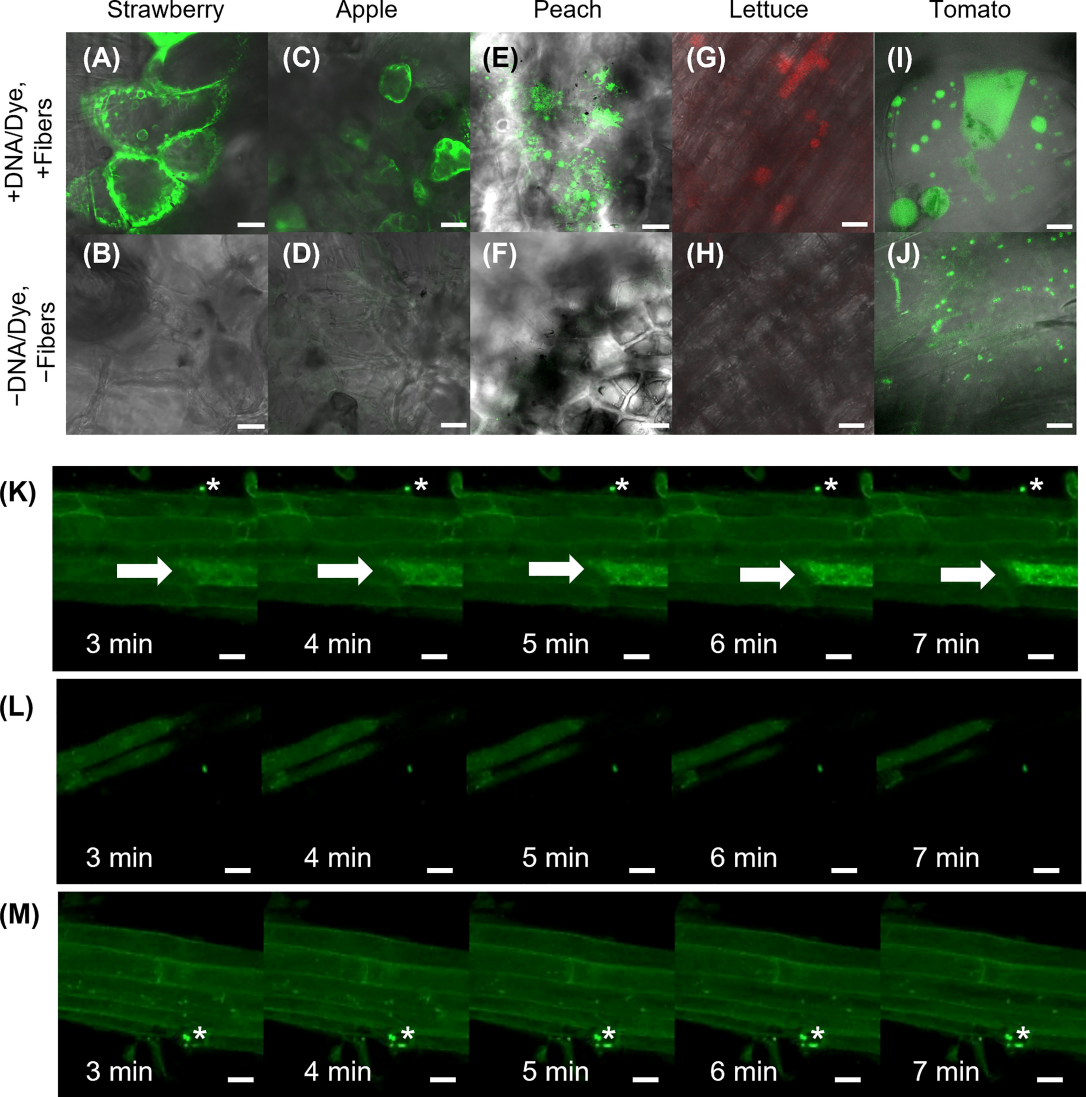
Application of VACNFs to curved organ surfaces. Images were generated using fluorescence confocal microscopy. Fluorescein dye was delivered to store-bought strawberry **(A)**, apple **(C)**, and peach **(E)** via fibers with 35 μm pitch with the on-plant method. **(G)** TAMRA dye was delivered to lettuce roots using the on-plant method with fibers with 10 μm pitch. **(I)** DNA (p35S:AALP:GFP/pUC) was delivered to store-bought tomato fruit and GFP fluorescence was imaged 40 h after delivery via on-plant method with fibers with 20 μm pitch. The area shown is approximately one large cell. Panels **(B, D, F, H,** and **J)** show no-treatment controls of plant tissue for panels **(A, C, E, G,** and **I)**, respectively. **(K–M)** DNA encoding FlincG was delivered to Arabidopsis roots via VACNFs with 35 μm pitch (on-chip method), and expression was assessed after 36 h. **(K)** 36 h after FlincG delivery, Arabidopsis roots were treated with Pep1 peptide (panels show different time points after exposure to elicitor Pep1 (20 nM)) (+DNA-FlincG, +fibers, +Pep1). The white arrow points to the transformed cell with signal increasing over time. **(L)** 36 h after FlincG delivery, Arabidopsis roots were treated with water (panels show different time points after exposure to water, which does not trigger production of cGMP) (+DNA-FlincG, +fibers, +water). **(M)** Arabidopsis roots with fibers without biosensor delivery treated with Pep1 (−DNA, +fibers, +Pep1). Scale bars are 20 μm. **(A–F)** are single images (0.5 μm thick) captured via confocal microscopy. **G, H** are maximum projections of 118 μm z-stacks and **I-M** are maximum projections of 40 μm z-stacks. * denote regions with fibers.

Previously, VACNFs were made to have the approximate diameter of aphid stylets (Davern et al., 2016) (**Figure 1**). These original nanofibers had a base diameter of 500 nm and tapered to 100 nm at the tip. Additionally, they had a lateral spacing (pitch) of 20 μm, to minimize the number of nanofibers penetrating individual epidermal cells in *Populus* leaves. We designed additional fiber arrays with longer (35 μm) or shorter (10 μm) pitch, reasoning that those additional geometries may be useful for a broad array of plant cells of various sizes and shapes of plant cells (**Figures 1C, D**). In this study, fibers with 35 or 20 μm pitch were used for large Arabidopsis epidermal cells that have a jigsaw-like appearance and do not have a uniform shape; cells are 20 to 50 wide μm and can be as long as 100 μm. These fibers were also used for dye delivery in peaches, strawberries, and apples. Fibers with shorter pitch (10 μm and 20 μm) were used for tomato fruit, and leaves of poplar, lettuce, and *N. benthamiana*. All fibers were 10-25 μm in length and all designs were successful in transforming plants (**Figures 1**–**6**).

### 3.2 On-plant VACNF-mediated transformation of Arabidopsis leaves with a small plasmid

To test the applicability of VACNFs for transient transformation, we first employed a green fluorescent protein (GFP) fusion to the Arabidopsis aleurain-like protein (AALP) that transits through the endomembrane system and targets vacuoles (Sohn et al., 2003; Kim et al., 2005; Kang et al., 2014). We used a small (~3000 bp) pUC-based vector (used previously for protoplast transformation) and the VACNF on-plant method on excised leaves (**Figure 1A**). The accumulation of AALP:GFP was detected by confocal microscopy in Arabidopsis leaves 48 h after transformation (**Figure 2A**). We quantified success of delivery by comparing measurements of fluorescence that resulted from delivery of the vector (+DNA-AALP:GFP, +fibers) and the various controls (−DNA, +fibers; +DNA-AALP:GFP, −fibers (flat side of the chip); and −DNA, −fibers (no treatment)). GFP signal was present only in leaf areas in which plasmid was delivered *via* fibers (+DNA-AALP:GFP, +fibers) (**Figures 2A–E**) and was not observed in controls. Background signal in **Figure 2** mainly comes from the stomatal guard cells, as indicated by asterisks, which were excluded from the analysis. When imaging the −DNA, +fibers control, fields of view were selected such that fibers (which are autofluorescent) were present (**Figure 2B**). These data show successful VACNFmediated transformation of Arabidopsis leaves, resulting in expression of a fluorescent reporter at levels easy to image by florescence microscopy.

### 3.3 On-plant and on-chip methods of VACNF-mediated transformation with a large plasmid

To further demonstrate the utility of the VACNF chips, we tested if a large plasmid encoding yellow fluorescent protein (YFP) could be used to transform leaves that were detached from plants. We used fibers with the on-plant method to transform Arabidopsis leaves with pUBQ_10_:YFP carried on the ~11,000 bp Gateway plasmid, usually used for *Agrobacterium*-mediated transformation (Michniewicz et al., 2015) (**Figure 3**). The leaves to which the plasmid was delivered *via* fibers (+DNA-YFP, +fibers), had significantly greater signal in the YFP channel than the controls (−DNA, +fibers; +DNA-YFP, −fibers (flat side of the chip); −DNA, −fibers (no treatment)) (**Figure 3I**). Similar to a small plasmid, the use of a large binary vector resulted in easily detectable reporter fluorescence. Background signals observed in **Figure 3** again mainly came from the guard cells, as indicated by the asterisks in the figure. In this experiment, we also compared the on-plant and on-chip methods of VACNF-mediated impalefection. There was no observable difference in the expression pattern of YFP in leaves transformed (**Figures 3A, B**). Additionally, there was no significant difference in measured fluorescence values for the on-chip and on-plant methods (**Figure 3I**). Therefore, we used the on-plant method in most subsequent experiments. Accounting for various factors, including where force was applied to the chip as well as the number of fibers that penetrated the leaf tissue, observable expression was detected in different areas within the 3mm×3mm area of leaf tissue under the chip. As the leaf surface is not completely flat, not all cells came into contact with the chip. These experiments show that detached leaves can be transformed with a large plasmid using VACNFs.

### 3.4 VACNF-mediated transformation of different plant species

To further substantiate the applicability of VACNF chips, the on-plant method was applied to excised poplar leaves to deliver DNA encoding AALP:GFP in the pUC vector. Arabidopsis leaves served as a positive control in this experiment to show that the quality of DNA was suitable for delivery and expression. GFP signals were observed in impaled regions 48 h post-delivery in Arabidopsis and poplar leaves (**Figures 4**). The fibers were also used to successfully deliver p35S:mCherry/pUC vector to lettuce (*Lactuca sativa* cv. Salinas) and *Nicotiana benthamiana* leaves attached to plants (**Figures 5A, B**). These results show that nanofibers can deliver DNA to different plants for the production of encoded proteins.

### 3.5 Comparison of VACNF and *Agrobacterium*-mediated transient transformation

*Agrobacterium* is commonly used for transformation of intact plants. Therefore, we compared expression of mCherry resulting from *Agrobacterium*-mediated transformation, using pCambia binary vector, to our VACNF-mediated delivery of p35S:mCherry/pUC in lettuce and *N. benthamiana* leaves attached to plants (**Figure 5**). mCherry fluorescence in lettuce leaf was much higher using VACNF-mediated impalefection than was found using agroinfiltration (**Figures 5A, C, E**). In contrast for *N. benthamiana*, which is highly amenable to transient transformation with *Agrobacteria*, mCherry fluorescence was higher after agroinfilitration than after imaplefection, as observed by microscopy (**Figures 5B, D, F**) and quantified (**Figure 5G**).

For the experiments described above with lettuce, it was necessary to optimize the timing of observations after VACNF-mediated transient transformation. In contrast to other plant species, we did not detect fluorescence in lettuce 2 d after transformation either *via* VACNFs or *Agrobacteria*. Wroblewski et al. (2005) previously noted that the highest transient expression occurred in lettuce 4-5 d after agroinfiltration. We detected mCherry signals 3-5 d after transformation of lettuce with both methods, with highest signal at 4 d. In *N. benthamiana* fluorescence was highest 3 d post transformation. Our results stress the importance of optimalization of VACNFs protocols for different plants, which may require different times for transgene expression after impalefection. The experiments with lettuce show that VACNFs can be a superior method for plant species and tissues recalcitrant to *Agrobacterium*-mediated transformation.

### 3.6 VACNFs may be useful for cargo delivery and transformation of different plant organs

Curved organs present a challenge for transient transformation using VACNFs, where having a relatively flat surface to tap the chip with nanofibers into the tissue is an advantage. Nevertheless, to establish broader applicability of the method, we tried various tissues, including hypocotyl, roots and fruit. While we did not get successful transformation of hypocotyls, we did have a few positive results with other curved organs. We found that it was useful as a first test to deliver a fluorescent dye (e.g., fluorescein or TAMRA) to the sample organ, as demonstrated by Davern et al. (2016). This permits a rapid assessment of whether the mechanics of the delivery method will work. In this way, we showed that VACNF arrays could successfully deliver dyes into fruit (store-bought strawberries, apples, and peaches) and lettuce roots (**Figures 6A–H**). In addition, we were able to transform tomato fruit with p35S:AALP:GFP/pUC using the on-plant method (**Figure 6I**). Specifically, after 40 h, GFP signals were observed in the tomato fruit pericarp when the chip contact area was excised from the fruit. We did not see significant GFP signals in the epidermal (skin) layer. For Arabidopsis roots, we used the on-chip method to deliver FlincG, a fluorescence-based reporter for cGMP. After delivery of the construct, we treated the roots with Pep1, a plant peptide that activates signaling, to induce cGMP, and monitored signal over time. We captured images of a transformed cell that showed increased fluorescence signal over time in response to Pep1 (**Figure 6K**), similar to what was previously reported (Ma et al., 2012). Controls for this experiment (+DNA-FlincG, +fibers, +water; and −DNA, +fibers, +Pep1) did not show an increase in green fluorescence over time (**Figures 6L, M**). Thus, delivery of the biosensor and application of an elicitor are necessary for increased fluorescence signal over time. This experiment indicates that a functional probe can be expressed in cells transformed using VACNF.

## 4 Discussion

In this work, two different VACNF chip impalefection protocols (on-plant and on-chip, **Figure 1**) were established for transient transformation of leaves, roots and fruit in a number of plant species (**Figures 2**–**6**). VACNF chips can be used on intact plants or on organs detached from plants. This raises the possibilities of a number of interesting applications, such as studying the impact of environmental perturbation on reporter gene activity. Both the on-plant and on-chip methods rely on the fibers to make small punctures in the cell wall and membrane through an impulse force resulting from the tapping of the tweezers on the back of the chip. Using the fibers in this manner is minimally invasive and was not found to be toxic or damaging to the plant (Davern et al., 2016). Importantly, these methods do not require fibers to be surface functionalized, unlike other existing methods (Demirer et al., 2019; Jat et al., 2020; Zhang et al., 2022b), and the fibers can be used to deliver different sized vectors. With the exception of hypocotyls, all plant organs that we tested could be transformed using VACNFs: leaves (Arabidopsis, poplar, lettuce, and *N. benthamiana*), roots (Arabidopsis) and fruit (tomato; **Figures 2**–**6**). Importantly, VACNF-mediated transformation may be successfully used in a species, such as lettuce, for which Agrobacterium-mediated transient transformation is inefficient (**Figure 5**).

**Figure 7:**
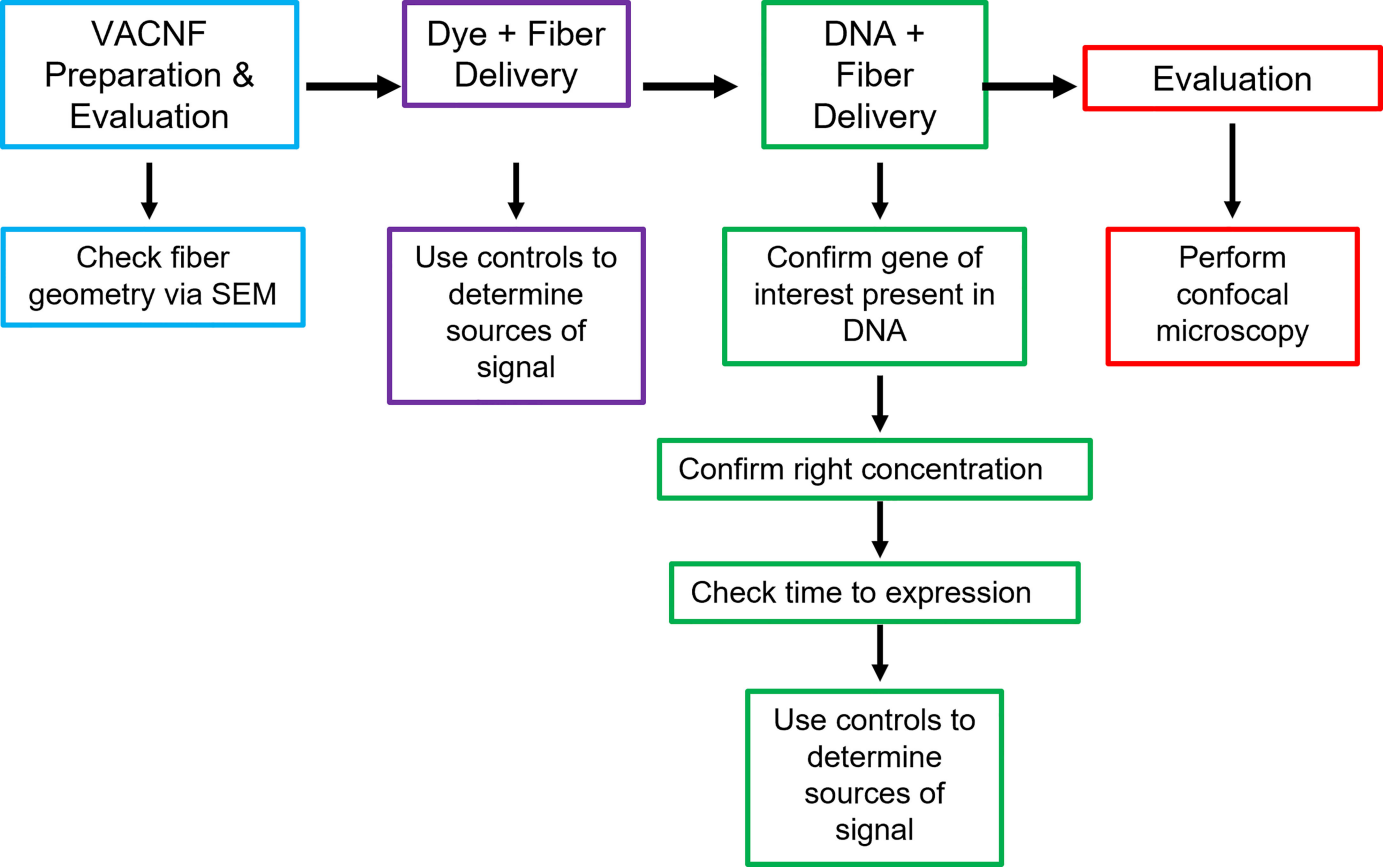
Workflow for VACNF mediated transient transformation of plants. When using the VACNFs for delivery of small molecules, there are a few problems that can arise. The first step in troubleshooting is to check the geometry of the fibers using scanning electron microscopy to ensure that the fibers are straight and have a sharp tip. After confirming the fiber geometry, the next step is to make sure that you are able to successfully deliver dye into your plant/organ of choice. For this purpose, place a 1 μL droplet on the plant surface and use the fibers to deliver the dye, checking for delivery with the microscope. With this step and all other steps moving forward, it is important to use appropriate controls (−dye,−fibers; −dye, +fibers; and +dye,−fibers), to be confident that you are getting signal from dye delivery. If both steps are successful, the next step would be to ensure that your gene of interest is present in your plasmid, you are using the right concentration of DNA, and you are waiting the right amount of time after delivery to check expression. The last step is to check expression using confocal microscopy.

In this study, VACNFs were produced at Oak Ridge National Laboratory CNMS through their user program, using previously described protocols (Melechko et al., 2005; Melechko et al., 2009). These publications also provide an overview and workflow of the fabrication of nanofiber arrays. VANCFs can also be produced at university clean rooms with direct current plasma enhanced chemical vapor deposition machines (Liu et al., 2010; Salmeen et al., 2015). A workflow summarizing important design, quality-control and operational steps for impalefection is provided in **Figure 7**. Successful DNA delivery with the VACNFs in plants depends upon on the geometry of the fibers and the design of the chips. The fibers used herein were straight with a tapering diameter such that the tip was less than 200 nm and resembled an aphid stylet. It was found that sharp tips with smaller diameters were less damaging than blunt tips in Arabidopsis leaf epidermal cells (Forouzesh, 2012; Forouzesh et al., 2014). In their study, Forouzesh et al. (2013) used single tips (or a single fiber equivalent) for nanoindentation studies to quantify the failure stress of cell walls. If the fibers are bent, they are unable to make direct contact with the leaf tissue, hence unable to impale cells and achieve delivery. Straightness of fibers is one of the first things to check if VACNF-mediated transformation fails (**Figure 7**). Pitch is another consideration, although in our experience it did not impact delivery success. The chips used in our experiments were designed to limit the number of fibers penetrating each cell (limit 1-2 fibers) by varying the pitch. Another factor that did prove critical for protein expression after VACNF-mediated impalefection is the timing. Most fluorescent markers were detected after 2 d. However, in lettuce, expression took longer, 4 d (**Figures 2**–**6**). The fluorescent markers encoded by VACNF-delivered DNA were detected in expected localizations: endomembrane compartments and vacuoles for AALP:GFP (Sohn et al., 2003; Kim et al., 2005; Kang et al., 2014), cytosol and nuclei for free mCherry and YFP (e.g. Kang et al. (2014)).

Both large (binary vectors utilized for *Agrobacterium*-mediated transformation) and small vectors (previously used for protoplast transformations) can be successfully delivered and expressed in plants (**Figures 2**–**6**). Smaller plasmids are easier to manipulate and tend to have a higher transient transformation efficiency in protoplast transfections and particle bombardment than the larger vectors used for *Agrobacterium*-mediated transformations (Sainsbury et al., 2009). With fiber delivery, results using larger and smaller vectors were similar. Therefore, constructs designed for use with *Agrobacterium* can be successfully used with VACNFs.

Nanomaterials are increasingly being used for transient transformation. The most recent methods of nanoparticle-mediated transformation or delivery of nucleic acids to plants have relied upon using pressure infiltration to deliver nanoparticles into plants *via* a needleless syringe (Zhang et al., 2019; Demirer et al., 2019; Izuegbunam et al., 2021; Zhang et al., 2022). Together, these nanoparticle-mediated methods have enabled delivery of nucleic acids and other molecules into Arabidopsis, tobacco, onion, maize, wheat, rapeseed, mustard, carrot, cotton, tomato, spinach, arugula, watercress, cowpea, chamomile, barley, and moss (Kumar et al., 2020; Jat et al., 2020; Izuegbunam et al., 2021). Roots can take up nanoparticles through passive diffusion and plant leaves can easily be transformed with passive infiltration of nanoparticles entering through the stomata (Hubbard et al., 2020). To the knowledge of the authors, only one study previously used nanoparticles to transform other parts of a plants that were not leaves or roots. Specifically, in Izuegbunam et al. (2021), the authors used needleless syringes to deliver nanorods with anchored DNA to developing seed tissues in Arabidopsis, field mustard, barley, and wheat. This method, like the aforementioned nanomaterial-mediated delivery of DNA (Demirer et al., 2019; Jat et al., 2020; Zhang et al., 2022b), requires the conjugation of DNA to nanorods and subsequent release of DNA once inside cells.

In summary, we have demonstrated the utility of using vertically aligned carbon nanofibers to overcome the challenges of crossing plant cell walls and plasma membranes to deliver DNA into cells. There is no release of cargo necessary, since nanofibers facilitate delivery through mechanical means into the plant, probably by forming transient channels around the fibers through which material can enter cells (Davern et al., 2016). The level of transgene expression readily allowed detection of several fluorescent reporters. Previously, VACNFs were successfully used to deliver proteins to plants cells (Davern et al., 2016). In that study dextran and proteins were detected in plant cells as early as 5 minutes after introduction *via* VACNFs. Labeled peptides entered vascular tissues which allowed their distribution throughout the plant (Davern et al., 2016). In the present work, we have demonstrated the value of VACNF chips in delivering DNA into leaves, roots, and fruit, with the minimally invasive impalefection allowing subsequent expression of the intended gene products. Looking forward, VACNF chips could be utilized to deliver multiple types of biomolecules together (e.g., gene editing reagents) and for stable transformation *via* delivery to a meristem, plant cell culture, or plants undergoing early organogenesis.

## Data availability statement

The original contributions presented in the study are included in the article/supplementary material. Further inquiries can be directed to the corresponding authors.

## Author contributions

JM, JJ, JG, RS, and JM-F conceived the research and designed the experiments. JM, SR, and DH designed and fabricated the carbon nanofibers. JM and JJ performed the experiments and analyzed the data. JM and JJ wrote the manuscript with input from JG, RS, SR, DH, and JM-F. All authors contributed to the article and approved the submitted version.

## Funding

This work has been funded by the Bioimaging Science Program, U.S. Department of Energy, Office of Science, Biological and Environmental Research, DE-SC001914 and the United States Department of Agriculture, 2021-67013-34835. JMM was supported by the United States Department of Agriculture: National Institute of Food and Agriculture: Agriculture and Food Research Initiative Predoctoral Fellowship 2021-67034-35167.

## Acknowledgments

We thank Dr. Inhwan Hwang (Pohang University of Science and Technology) for CaMV p35S: AALP:GFP DNA and Dr. Jiyoung Lee (Korea Research Institute of Bioscience and Biotechnology) for p35S-mCherry constructs. We thank John Zdenek for assistance with plant growth and maintenance. We thank Mindy Clark from Oak Ridge National Laboratory and Dr. Gail Taylor from the University of California, Davis for *Populus trichocarpa* (Nisq-1 genotype). Nanofiber arrays were fabricated at the CNMS, which is a Department of Energy Office of Science User Facility (Proposal ID : CNMS2019-103). Support from CNMS is awarded through a peer-reviewed proposal system and is provided at no cost to successful applicants who intend to publish their results (http://www.cnms.ornl.gov/user/becoming_a_user.shtml). We thank the CNMS for assistance with the production of nanofiber arrays. We thank Dr. John Caughmen, Dr. Timothy McKnight, and Travis Bee for critical discussion about experimental design.

## Conflict of interest

The authors declare that the research was conducted in the absence of any commercial or financial relationships that could be construed as a potential conflict of interest.

## Publisher’s note

All claims expressed in this article are solely those of the authors and do not necessarily represent those of their affiliated organizations, or those of the publisher, the editors and the reviewers. Any product that may be evaluated in this article, or claim that may be made by its manufacturer, is not guaranteed or endorsed by the publisher.

## Abbreviations

AALP, Arabidopsis aleurain-like protein; CNMS, Center for Nanophase Materials Sciences; VACNFs, Vertically aligned carbon nanofibers.
